# 

**Published:** 1844-01

**Authors:** 


### BRITISH AND FOREIGN

### MEDICAL REVIEW

OR

### QUARTERLY JOURNAL

OF

### PRACTICAL MEDICINE AND SURGERY

STATE OF LOUISIANA CHARITY HOSPITAL * MEDICAL LIBRARY. * JUSTICE
LIBRARY OF THE
Orleans Parish Medical Society

EDITED BY

JOHN FORBES M.D. F.R.S. F.G.S.

---

VOL. XVII

JANUARY—APRIL 1844

LONDON

JOHN CHURCHILL PRINCES STREET SOHO

MDCCCXLIV.

C. AND J. ADLARD, PRINTERS, BARTHOLOMEW CLOSE.

### BOOKS RECEIVED FOR REVIEW.

1. On Ankylosis, or Stiff Joint; a practical treatise on the contractions and deformities resulting from diseases of the joints. By W. J. Little, M.D.—London, 1843. 8vo, pp. 149. 8s. 6d.

2. On the Arrangement and Nomenclature of Mental Disorders; a Prize Essay. By H. J. Johnson, M.D.—London, 1843. 8vo, pp. 33. 1s. 6d.

3. Removal of Dropsical Ovaria entire, by the large Abdominal Section. By H. D. Walne, Surgeon. (From the Medical Gazette.)—Lond. 1843. 8vo, pp. 11. 3s. 6d.

4. Principles of Forensic Medicine. By W. A. Guy, M.B. &c. Part I.—Lond. 1843. 8vo, pp. 184. 4s.

5. The Vital Statistics of Sheffield. By G. C. Holland, M.D.—London, 1843. 8vo, pp. 263.

6. Practical Directions for the Preparation of Aërated Waters, &c. By R. Venables, A.M., M.B.—Lond. 1843, 12mo, pp. 110. 4s.

7. Some Account of the Epidemic Scarlatina which prevailed in Dublin from 1834 to 1842 inclusive. By Henry Kennedy, A.B, M.B. &c.—Dublin, 1843. Small 8vo, pp. 213.

8. Tic Douloureux, or Neuralgia Facialis, &c. By R. H. Alnatt, M.D. Second Edition[?].—Lond. 1843. 8vo, pp. 223. 5s.

9. Glossology; or, the additional means of Diagnosis of Disease to be derived from indications and appearances of the Tongue. By B. Ridge, M.D. (Read before the Senior Society of Guy's Hospital, Nov. 4, 1843.)—Lond. 1844. 8vo, pp. 88, with Engravings.

10. A Pathological and Philosophical Essay on Hereditary Diseases; with an Appendix, on Intermarriage, &c. By J. H. Steinau, M.D.—Lond. 1843. 8vo, pp. 52. 3s. 6d.

11. Lectures on the Principles and Practice of Physic, delivered at King's College, London. By T. Watson, M.D., &c. In two vols.—Lond. 1843. 8vo, pp. 830, 812. 34s.

12. Animal Physiology (Popular Cyclopædia of Natural Science). By Wm. B. Carpenter, M.D.—London, 1843. 8vo, pp. 579. 10s.

13. A Practical Treatise on Organic Diseases of the Uterus; the Fothergillian Prize Essay for 1843. By J. C. W. Lever, M.D. —London, 1843. 8vo, pp. 240. 9s.

14. Elementary Instruction in Chemical Analysis. By Dr. C. R. Fresenius. Edited by J. L. Bullock.—London, 1843. 8vo, pp. 284. 9s.

15. Principles of Medicine. By C. J. B. Williams, M.D., F.R.S.—Lond. 1843. 8vo, pp. 390. 12s.

16. Pathological and Histological Researches in Inflammation of the Nervous Centres. By J. H. Bennett, M.D., F.R.S.E., &c.—Edinburgh, 1843. 8vo, pp. 83.

17. Report of the Commissioners appointed to take the Census of Ireland for the year 1841.—Dublin, 1843. Folio, pp. 887.

18. The British Journal of Homœopathy. Edited by J. J. Drysdale, M.D., J. R. Russell, M.D., and F. Black, M.D.—Edinb. 1843. Vol. I.

19. The Oculist's Vade Mecum: or complete practical system of Ophthalmic Surgery. By John Walker, Surgeon to the Manchester Eye Hospital.—Lond. 1843. Sm. 8vo, pp. 400. With woodcuts. 10s. 6d.

20. Elements of Natural Philosophy. By Golding Bird, M.D., F.L.S. Second Edition, enlarged.—London, 1844. 8vo, pp. 479. 12s. 6d.

21. An Anatomical Description of the Gravid Uterus and its contents. By the late William Hunter, M.D., &c. Second Edit. By Edward Rigby, M.D.—London, 1843. 8vo, pp. 75. With 8 plates. 5s.

22. Principles of Forensic Medicine. By W. A. Guy, M.B. Part II.—Lond. 1843. 4s.

23. The Surgeon's Vade Mecum. By R. Druitt, Surgeon. 3d Edit, with 95 Engravings.—Lond. 1843. 8vo, pp. 572. 12s. 6d.

24. The Physiology of Inflammation, and the Healing Process. By Benjamin Travers, F.R.S., &c.—London, 1844. 8vo, pp. 226. 7s.

25. A concise Exposition of Homœopathy, its Principles and Practice. By G. Newman, M.R.C.S.—London, 1844. 8vo, pp. 68.

26. A Manual of Medical Jurisprudence. By Alfred S. Taylor.—London, 1843. 8vo, pp. 679. 12s. 6d.

27. On Superstitions connected with the History and Practice of Medicine and Surgery. By T. J. Pettigrew, F.R.S.—Lond. 1844. 8vo, pp. 167. 7s.

28. A Dictionary of Practical Medicine. By J. Copland, M.D., F.R.S. Part IX. 4s. 6d.

29. The Sources of Physical Science: being an introduction to the study of Physiology through Physics. By Alfred Smee, F.R.S.—Lond. 1843. 8vo, pp. 246. 10s. 6d.

30. The Cold Water Cure, &c. By R. Beamish, Esq., F.R.S.—Lond. 1843. 8vo, pp. 100.

---

ERRATUM. In the review of Becquerel, in our last Number, by an oversight which we much regret, the words "grain" and "grains" are printed for "gramme" and "grammes" throughout the article.

